# Patterns of language and auditory dysfunction in 6-year-old children with epilepsy

**DOI:** 10.1080/03009730802635927

**Published:** 2009-04-24

**Authors:** Gunilla Rejnö-Habte Selassie, Ingrid Olsson, Margareta Jennische

**Affiliations:** ^1^Institute of Neuroscience and Physiology/Speech and Language Pathology, Sahlgrenska Academy at the University of GothenburgGothenburgSweden; ^2^Institute of Clinical Sciences/Paediatrics, Sahlgrenska Academy at the University of GothenburgGothenburgSweden; ^3^Department of Neuroscience/Speech and Language Pathology, Medical Faculty, Uppsala UniversityUppsalaSweden

**Keywords:** Auditory dysfunction, childhood epilepsy, dichotic listening, language dysfunction

## Abstract

In a previous study we reported difficulty with expressive language and visuoperceptual ability in preschool children with epilepsy and otherwise normal development. The present study analysed speech and language dysfunction for each individual in relation to epilepsy variables, ear preference, and intelligence in these children and described their auditory function. Twenty 6-year-old children with epilepsy (14 females, 6 males; mean age 6:5 y, range 6 y–6 y 11 mo) and 30 reference children without epilepsy (18 females, 12 males; mean age 6:5 y, range 6 y–6 y 11 mo) were assessed for language and auditory ability. Low scores for the children with epilepsy were analysed with respect to speech-language domains, type of epilepsy, site of epileptiform activity, intelligence, and language laterality. Auditory attention, perception, discrimination, and ear preference were measured with a dichotic listening test, and group comparisons were performed. Children with left-sided partial epilepsy had extensive language dysfunction. Most children with partial epilepsy had phonological dysfunction. Language dysfunction was also found in children with generalized and unclassified epilepsies. The children with epilepsy performed significantly worse than the reference children in auditory attention, perception of vowels and discrimination of consonants for the right ear and had more left ear advantage for vowels, indicating undeveloped language laterality.

## Introduction

Speech and language ability has previously rarely been comprehensively described in studies of cognitive abilities in children with epilepsy. According to Deonna (1) there are several situations in which a direct causal link between epilepsy and language disorder exists. Both language and the motor command of speech can be affected.

There are recent indications that epilepsy may be more common in language-impaired children than is generally known. A high proportion of Electroencephalogram (EEG) abnormalities and epileptic syndromes has been found in children with severe language impairment (2), and Sillanpää reported a high proportion of speech disorder in children with epilepsy (3). Speech and language difficulties are known to be associated with specific epilepsy syndromes, such as the Landau Kleffner syndrome (LKS) and benign childhood epilepsy with centrotemporal spikes (BCECTS) (1,4,5), but they have also been reported in focal epilepsy (6,7).

Recently, we described speech, language, and cognition in a multidisciplinary study of a regional cohort of preschool children with epilepsy without previously known learning disability, cerebral palsy, and/or autism (8). Low achievements were found in visuoperception and expressive verbal ability. The study included a heterogeneous group of children with epilepsy, and therefore questions were raised regarding the individual patterns of dysfunction with respect to the different types of epilepsy and sites of epileptiform activity. In the present study the performance of these children is presented individually, and the auditory function of the children is further analysed.

The following specific questions were asked:

- Do children with the same types of epilepsy or sites of epileptiform activity display deficits in the same speech, language, and communication domains, and do those with different types and localizations display different deficits?
- Which ear preference for auditory stimuli is found in children with partial and generalized epilepsy and with different locations of epileptiform activity?
- Is there a difference between the children with epilepsy and the reference children in terms of auditory attention, perception, discrimination, and ear preference?

## Method

### Participants

The regional cohort (8) of children with epilepsy and without previously known cerebral palsy, learning disability, or autism from the city of Göteborg and eight surrounding municipalities comprised 14 girls and 6 boys, aged 6 years to 6 years 11 months (mean age 6:5 years). Ten children had partial epilepsy with or without secondary generalization, three with focal epileptiform activity in the left hemisphere, three in the right hemisphere, one in the frontal lobes, and one with epileptiform activity alternating between the right and left temporal lobes; in two children, the interictal EEG was normal. Six children had primary generalized seizures; five of them had absence epilepsy and one myoclonic astatic epilepsy. Four children were allocated to the group ‘undetermined’ whether focal or generalized, one of whom had Landau Kleffner syndrome (LKS). All the children were receiving antiepileptic drug treatment. The median full-scale IQ (FSIQ) was 95, verbal IQ (VIQ) 103, and performance IQ (PIQ) 88. Two children had a previously unknown learning disability (FSIQ < 70). Four children were bilingual. All the children had normal hearing within speech frequencies, apart from one child with a hearing level of 25 dB due to a temporary infection. Thirty children without epilepsy, 18 girls and 12 boys, were used as a reference group. Mean age of the reference children was 6:5 years, range 6 years to 6 years 11 months. They were similar in terms of age, sex, place of living, and mono-/bilingualism (8).

### Assessment procedure

The assessment battery comprised a wide range of tests for speech, language, and auditory ability, presented in the same order for all the children. The measures used for these tests are shown in Table I. For an overview, the tests are grouped into domains.

1. *Domain of oral motor ability and articulation*. The neurolinguistic test battery (Nelli) (9) and the quantitative measures according to Jennische (10) were used to assess oral motor ability and articulation. Oral motor ability is divided into ‘positions’, finding positions with tongue and lips, and ‘movements’, meaning repeated sequences of oral movements. Articulatory positions is a test of the repetition of isolated sounds and syllables and of rapid sequences of equal syllables.
2. *Domain of phonology and literacy*. Articulatory patterns refer to phonology-driven sequences of varying syllables, words, and word combinations and non-words from the Nelli test (9). Phonology was assessed with a repetition task, including the children's representations of all Swedish phonemes. Emerging literacy was assessed with phoneme blending, a subtest of the Illinois Test of Psycholinguistic Abilities (ITPA) (11), and letter naming.
3. *Domain of grammar and semantics*. Expressive grammar was assessed with the grammar subtest of the ITPA, reflecting morphology. Receptive grammar was assessed with the Test for Reception Of Grammar (TROG), reflecting both morphology and syntax (12). Receptive vocabulary was assessed with the Peabody Picture Vocabulary Test (PPVT) (13).
4. *Domain of word retrieval and narrative ability*. For expressive vocabulary, the word retrieval subtest of the ITPA (retrieval within semantic categories) and Rapid Confrontation Naming (RCFN) (picture naming) were used (14). In RCFN, the time and number of mistakes were registered. In addition, the auditory analogy ITPA subtest was used. Narrative ability was assessed with the story retelling test of Nelli. The number of events missing from a total of ten was registered.
5. *Domain of pragmatic ability*. This domain was assessed with the Children's Communication Checklist (CCC) parental questionnaire (15). For the pragmatic composite measure, the total scores were given.
6. *Domain of auditory attention and memory*. In this domain, the auditory attention measure of the dichotic listening test (DL) is included (see below). Auditory short-term memory was assessed with the digit span test (ITPA).

**Table I.** Speech and language test battery for 6-year-old children with epilepsy and reference children.

Domain	Variables	Speech and language tests	Type of measure
1	*Oral motor function*:		
	positions	Nelli	Ordinal scale 0–5
	movements	Nelli	Ordinal scale 0–5
	*Articulation:* positions	Nelli	Ordinal scale 0–5
2	*Articulation:* patterns	Nelli	Ordinal scale 0–5
	*Phonology*	Word/sentence repetition	Ordinal scale 0–5
	*Emerging literacy*: phoneme blending	ITPA	Stanine 1–9
	*Emerging literacy*: letter naming	20 letters	No. of correct
3	*Grammar*: expressive morphology	ITPA	Stanine 1–9
	*Grammar*: receptive morphology and syntax	TROG	No. of blocks
	*Vocabulary:* receptive (semantics)	PPVT	Stanine 1–9
4	*Vocabulary*: expressive: word retrieval	ITPA	Stanine 1–9
	*Vocabulary:* expressive: rapid picture naming	Rapid Confrontation Naming (RCFN)	No. of mistakes; Time (seconds)
	*Vocabulary:* auditory analogy	ITPA	Stanine 1–9
	*Narratives:* story retelling	Nelli	No. of partial events, ordinal scale 0–5
5	*Pragmatics (communication)*	CCC	Pragmatic composite, raw scores
6	*Auditory short term memory*: digit span	ITPA	Stanine 1-9
	*Auditory ability:*		
	attention	Dichotic listening	Per cent correct, CV pairs
	perception level		Per cent correct, C, V, CV
	discrimination		^a^
	ear advantage		Laterality index

^a^=Number of consonants and vowels correctly repeated <10% of times given. C = consonants; V = vowels; CV = consonant-vowel syllables; ITPA = Illinois Test of Psycholinguistic Abilities; TROG = Test for Reception Of Grammar; PPVT = Peabody Picture Vocabulary Test; CCC = Children's Communication Checklist.

### Auditory attention, level of perception, discrimination, and ear advantage

A dichotic listening test (DL) (16) was used to assess auditory attention, auditory perception level, auditory discrimination, and ear preference, using 108 pairs of consonant-vowel (CV) syllables with randomly varying consonants /p, t, k, b, d, g/ and vowels /a, i, u/. Different CV syllables were presented simultaneously to both ears in a non-forced condition through sound-proof headphones (HD 200, Sennheiser, Tullamore, Republic of Ireland). The children were asked to repeat the pairs of syllables. The results were analysed with respect to the percentage of correctly repeated consonants, vowels, and whole CV syllables for each ear. The level of auditory attention was calculated as the percentage of simultaneously presented pairs of CV syllables correctly repeated. The level of auditory perception was calculated as the average percentage of all correctly repeated consonants, vowels, and CV syllables for each ear. As a measure of auditory discrimination, the number of consonants and vowels that were correctly repeated less than 10% of the times given to each ear was used. The value of vowels could vary between 0 and 3 for each ear (3 corresponding to the lowest discrimination ability), and the value of consonants could vary between 0 and 6 (6 being the lowest ability). The inability to discriminate between the separate consonants and vowels could thus be quantified. The laterality index for consonants, vowels and CV syllables was calculated as (RE + LE)/(RE-LE)×100. Right ear advantage (REA) was established when the laterality index was equal to or greater than +5; left ear advantage (LEA) when it was equal to or below −5; no ear advantage (NEA) when the laterality index was between +5 and −5.

### Statistical analysis

The results representing a low score were defined for each speech and language test. A result of the stanine value 1 was defined as a low score for the subtests of the ITPA, and a result corresponding to the 10th percentile or lower in norms for Swedish children was defined as a low score for the TROG and the PPVT. For tests where no Swedish norms were available, the results of the reference group were used as norms. Thus, a result equivalent to, or lower than, the score that corresponded to the 10th percentile of the reference group with the lowest achievements was defined as a low score. The number of low scores within each language domain were counted and presented for each child with epilepsy, together with the type of EEG abnormality, site of epileptiform activity, FSIQ, VIQ and PIQ, and ear preference (Table II). The percentage of the children with epilepsy and the reference children with at least one low score within each domain are given. The average of low scores for speech and language tests for both groups was calculated. Comparisons between the children with epilepsy and the reference group were made for the assessments of the DL test: auditory attention, auditory perception level, and auditory discrimination (Mann-Whitney U-test). Ear preference was compared using the chi-square test. Statistical significance was defined as a *P*-value of <0.05, two-tailed test.

**Table II.** Epilepsy group. Results presented individually for each child: number of variables with low scores (≤ the score which was reached by the 10th percentile of the reference group with the lowest achievements within each domain, intelligence, epilepsy variables and ear preference.

Child code number	Type of seizures/syndrome	Type and site of epileptiform activity	Oral motor ability/articulation (a total of 3 variables)	Phonology/literacy (a total of 4 variables)	Grammar/semantics (a total of 3 variables)	Word retrieval/narrative ability (a total of 4 variables)	Communicative ability (a total of 1 variable)	Auditory attention/memory (a total of 2 variables)	Number of subtests with low scores/child	FSIQ	VIQ	PIQ	Ear adv CV	Ear adv vow	Ear adv cons
6	Partial	Left parieto-temporo-occipital	3	3	3	2	1	1	13	66	90	46	LEA	LEA	LEA
7	Partial	Left, centro-parietal	2	4	1	2	0	1	10	80	80	85	REA	REA	REA
10^a^	Partial	Left, medio-temporal	1	2	2	2	0	1	8	77	85	74	REA	REA	REA
4	Partial	Right, temporal	1	2	0	3	0	2	8	74	89	63	LEA	LEA	LEA
8	Partial	Right, parietal	1	2	0	0	0	1	4	90	96	85	LEA	LEA	LEA
12	Partial BCECTS	Right	2	1	0	0	0	0	3	114	116	107	REA	NEA	REA
1	Partial	Alternating temporal	3	2	0	2	0	0	7	84	97	72	REA	REA	REA
5	Partial	Frontal	2	1	0	0	0	1	4	104	113	93	REA	REA	REA
15^a^	Partial	EEG normal	1	0	3	5	1	0	10	112	107	102	REA	REA	REA
19	Partial	EEG normal	1	0	0	0	0	0	1	112	110	111	REA	REA	REA
3	Gen/CAE	3 Hz sp-w	2	1	2	2	0	1	8	93	104	81	LEA	LEA	LEA
9^a^	Gen/CAE	3 Hz sp-w	1	0	0	0	0	0	1	106	109	100	REA	REA	NEA
14^a^	Gen/CAE	3 Hz sp-w	1	0	1	2	0	0	4	92	101	84	NEA	REA	NEA
16	Gen/CAE	3 Hz sp-w	0 ^c^	0 ^c^	0	0	0	1	1	104	123	81	REA	REA	REA
20	Gen/CAE	3 Hz sp-w	2	1	0	3	0	2	8	103	107	97	REA	REA	REA
13^d^	Gen/M-A	Poly sp-w	2	3	2	4	1	2	14	64	72	64	^c^	^c^	^c^
2	Undeterm	Poly sp-w	1	2	0	1	0	2	6	97	102	92	LEA	LEA	LEA
11	Undeterm/LKS	Multifocal	1 ^b^	3 ^b^	1	4	0	2	11	90	91	91	REA	REA	REA
17	Undeterm	Left frontal	1	2	0	0	0	1	4	106	112	97	LEA	LEA	NEA
18	Undeterm	Sp-slow w	1	0	0	0	0	0	1	128	135	113	REA	NEA	NEA
Epilepsy group%			95	70	40	60	15	65							
Reference group%			50	53	37	27	13	27							

^a^=Bilingual. ^b^=Refused to perform some tasks because of severe difficulty. ^c^=Not possible to assess due to a ligament of the tongue. BCECTS = benign childhood epilepsy with centrotemporal spikes; CAE = childhood absence epilepsy; cons = consonants; CV = consonant-vowel syllables; Ear adv = ear advantage; EEG = electroencephalogram; FSIQ = full-scale IQ; Gen = generalized; LEA = left ear advantage; LKS = Landau Kleffner syndrome; M-A = epilepsy with myoclonic astatic seizures; NEA = no ear advantage; PIQ = performance IQ; REA = right ear advantage; sp = spikes; undeterm = undetermined whether focal or generalized; VIQ = verbal IQ; vow = vowels; w = waves. ^d^=Not possible to assess due to poor cooperation.

### Ethics

Ethical permission for the study was given by the local ethics committee, while written informed consent to participate was received from all parents.

## Results

### Domains of speech and language dysfunction

Almost all the children with epilepsy had low scores for at least one of the variables within the oral motor domain (19/20, 95%) (Table II). In the domain of phonology and literacy, 14/20 (70%) of the children had low scores for one or more variables. In the domain of grammar and semantics, 8/20 children with epilepsy (40%) had low scores, while, in the domain of word retrieval and narrative ability, 12/20 (60%) obtained low scores. In the domain of pragmatic ability, only three children with epilepsy (15%) were reported to have difficulties, while, in the domain of auditory attention and memory, 13/20 (65%) obtained low scores. The comparable percentages for the reference children are given in Table II. On average, the children with epilepsy had low scores in 6.2 measures, as opposed to 3.0 measures for the reference group.

### Speech and language dysfunction, epilepsy variables, and intelligence

In children with partial epilepsy, the following pattern was found: all three children (code nos. 6, 7, and 10) with a left hemispheric site of epileptiform activity had low scores in one or more tests in all the language domains except pragmatics, indicating broad-based language disorders of varying degrees. In contrast, the three children with a right hemispheric site of epileptiform activity (code nos. 4, 8, and 12) did not obtain low scores in the domain of grammar/semantics, and only one of them obtained low scores for word retrieval/narrative ability.

In children with generalized epilepsy, the pattern of dysfunction was less uniform: one child with myoclonic astatic epilepsy (code no. 13) had a dysfunction in all domains, two of the children with absence epilepsy (code nos. 9 and 16) had a dysfunction in only one domain, while the others (code nos. 3, 14, and 20) had varied patterns of dysfunction.

The child with Landau Kleffner syndrome (LKS) (code no. 11) had low scores in all domains except pragmatic ability, and the dysfunction confirmed a diagnosis of expressive language impairment. This child refused to perform some of the tests because of additional severe difficulties with articulation and stuttering.

Despite low scores for speech and language measures, 17/20 children had a higher VIQ than PIQ, with a VIQ of >85 in all but 2 and a PIQ of <85 in 8. One of the two children with an FSIQ of <70 (code no 6) had a VIQ of 90 and a PIQ of 46, indicating a visuoperceptual deficit rather than a general learning disability (Table II).

### Ear advantage and epilepsy variables

No clear pattern of association with type of epilepsy, site of epileptiform activity, and ear preference was observed. LEA (right hemispheric dominance) was found in the partial, generalized, and unclassified types of epilepsy (Table II).

### Group comparisons of the dichotic listening test

The children with epilepsy obtained statistically significantly lower scores than the reference group in the auditory attention measure of the DL test (*P =* 0.018) (Table III). In three measures of the DL test, the children with epilepsy obtained significantly lower scores than the reference children, indicating a less dominant left hemisphere for the analyses of speech sounds: the level of auditory perception of vowels presented to the right ear (*P =* 0.019), the discrimination of consonants presented to the right ear (*P =* 0.015), and ear preference for vowels (*P =* 0.042) (Table III), the latter presented in our previous study (8). Of 19 children with epilepsy, 6 (32%) had a clear LEA (right hemispheric dominance—code no. 17 had NEA only for consonants) and another 4/19 children (20%) had NEA (no hemispheric dominance) in one or more of the CV syllables, vowels, or consonants (Table II). In the reference group, only 4 of 29 children (14%) had a clear LEA, while 13/29 (45%) had NEA in one or more of the CV syllables, consonants, or vowels. As a result, LEA was more common in the children with epilepsy (8).

**Table III.** Auditory attention, perception and discrimination measured with the dichotic listening test in 19 6-year-old children with epilepsy and 29 reference children. Comparison between groups (Mann-Whitney U-test).

	Epilepsy group			Reference group			
Variables	*n*	Median	Min-max	*n*	Median	Min-max	*P*
Auditory attention							
% correct CV pairs	19	1	0–13	29	3	0–36	0.018
Auditory perception level							
% consonants right ear	19	35	14–53	29	41	20–68	0.126
% consonants left ear	19	33	14–51	29	29	20–64	0.673
% vowels right ear	19	59	9–91	29	72	42–96	0.019
% vowels left ear	19	52	16–86	29	57	29–91	0.435
% CV right ear	19	28	7–50	29	31	7–69	0.167
% CV left ear	19	21	9–46	29	20	11–57	0.941
Auditory discrimination							
*n*<10% correct consonants right ear	19	2	0–4	29	1	0–4	0.015
*n*<10% correct consonants left ear	19	2	0–4	29	2	0–4	0.895
*n*<10% correct vowels right ear	19	0	0–2	29	0	0–1	0.796
*n*<10% correct vowels left ear	19	0	0–1	29	0	0–2	0.292

*n*=number; min = minimum value; max = maximum value; CV = consonant–vowel.

## Discussion

In this cohort of 6-year-old children with epilepsy and previously expected normal development, recruited from a regional sample, the majority obtained low scores for several aspects of speech and language. Almost all the children with epilepsy had low scores in the domain of oral motor ability/articulation, and around two-thirds of the children obtained low scores within the domains of phonology/literacy and auditory attention/memory. Different localizations of epileptiform activity resulted in different patterns of dysfunction. In children with partial epilepsy with a left-sided focus, the dysfunction was most pronounced. Furthermore, children with generalized seizures also displayed a dysfunction in a variety of speech and language areas. Tests of dichotic listening revealed lower scores in auditory attention, perception level, and discrimination, and unclear or different laterality for language in the children with epilepsy compared with the reference group.

Oral motor dysfunction, as found in our study group, has previously been reported in many children with epilepsy. However, it is unclear whether this is an effect of medication or of the epileptic activity (17), and our study group was too small to answer this question. In addition, stuttering in children with epilepsy has recently been highlighted (18,19). We also found that stuttering was an obvious impediment in the child with LKS.

Phonological deficits have mainly been reported in children with BCECTS (1) but seldom in a group of children including other types of epilepsy, such as we found. The domain of phonology/literacy included tests of phoneme blending, letter naming, and non-word repetition, and low scores in may be predictors of subsequent reading and writing difficulties (20). Other studies have also reported reading and writing difficulties in children with epilepsy (1,21–23).

Deficits in general language-processing capacity is thought to underlie verbal expressive disorder, as in the domain of word retrieval/narrative ability and in auditory attention/short-term memory (17,20). Our results indicate that children with epilepsy have a general language-processing disorder, causing the deficits in these domains. However, assessment of auditory attention in small children is difficult to perform, as stated by Hugdahl (24). We used a procedure of simultaneous attention to competing stimuli, which has not previously been tested, and our results should therefore be interpreted with caution. However, they agree with the results reported by Svoboda, who stated that children with epilepsy often have difficulty listening to competing stimuli against a noisy background (17).

Focal seizures affecting brain areas subserving speech and language are thought to be linked to specific speech and language disability (1). In the present study, this was apparent in the children with a left hemispheric focus, who had more severe dysfunction. One child with left temporal partial epilepsy had a pattern of dysfunction compatible with severe expressive language disorder, indicating a possible direct effect of the epilepsy on speech and language. In addition, one child with a severe expressive disorder had LKS, known to be a predominantly receptive disorder. This indicates variability in symptoms in LKS. Dysfunction in various language domains was also present in some children with generalized seizures, indicating a general effect on language competence, which is not usually reported.

Our results for language laterality are not clearly linked to a particular type of epilepsy, such as those of Pisano et al., who found a lack of hemispheric specialization for phonological processing and impaired access to stored lexical knowledge in familial lateral temporal lobe epilepsy (25). Pecini and colleagues found that children with the expressive subtype of specific language impairment had a reduced specialization for language compared with age-matched controls (26). These findings are comparable to the expressive difficulties in our children with epilepsy, in which several measures revealed a different laterality for the reception and analysis of speech sounds compared with reference children of the same age. It is thus probable that the epileptic activity affects the development of language laterality in these children. However, we cannot exclude the possibility that AED treatment may have influenced this hampered development.

When assessing children with epilepsy, difficulty with attention and memory may affect the results, and this should be taken into consideration when choosing test instruments. The dysfunction in speech, language, and auditory ability found in our study group indicates potential difficulty with subsequent school achievements. The dysfunction in memory and attention may be an obstacle to general performance. Speech and language intervention and extra support for reading and writing acquisition may be needed, as well as additional instruction in the classroom and the opportunity to rest.

### Limitations

This study was performed on a small number of children and the results need to be replicated. The dichotic listening test is difficult to perform in 6-year-old children, as the attention ability is not yet fully developed in this age group. The results should be interpreted with caution, as there was great variation in the results for both groups.

## Conclusion

Six-year-old children with epilepsy and no previously known cerebral palsy, learning disability, or autism may have a dysfunction in all speech and language domains, in spite of verbal intelligence within the normal range. Children with a left temporal epileptic focus or the Landau Kleffner syndrome are those with the most extensive speech and language dysfunction, and partial epilepsy with either a left- or right-sided focus is more often associated with a dysfunction in phonology. Children with other epileptic foci and with generalized or unclassified epilepsy may also have a dysfunction in speech and language, although less specific. Unusual ear preference and poor auditory perception and discrimination indicate the hampered development of language laterality. The long-term consequences of epilepsy in school-age children need to be studied.
